# Supplementary material for: Evolutionary insights from profiling LINE-1 activity at allelic resolution in a single human genome
Source: EMBO J. 2023 Dec 18;43(1):6. doi: 10.1038/s44318-023-00007-y (PMC10883270; doi:10.1038/s44318-023-00007-y)
Supplement: Supplementary file 2 — Table EV1 [file 44318_2023_7_MOESM2_ESM.pdf]

Table EV1. A comparison of intact LINE-1s across GRCh38 and CHM genome assemblies. Counts of the number of LINE-1s that are intact, present but not intact (non-intact), and absent are shown. Only LINE-1s that are intact in one of the genomes in each comparison are counted - ‘NA's represent the categories where LINE-1s are intact in none of the genomes compared.

3-way comparison between GRCh38, CHM1 and CHM13

GRCh38-CHM1-CHM13		GRCh38 intact	GRCh38 non-intact	GRCh38 absent	Total
CHM1 intact	CHM13 intact	72	19	11	102
	CHM13 non-intact	15	12	2	29
	CHM13 absent	8	0	15	23
CHM1 non-intact	CHM13 intact	9	9	1	19
	CHM13 non-intact	15	NA	NA	NA
	CHM13 absent	0	NA	NA	NA
CHM1 absent	CHM13 intact	7	0	16	23
	CHM13 non-intact	1	NA	NA	NA
	CHM13 absent	29	NA	NA	NA
Total	SUM	NA	NA	NA	196

Total LINE-1	454
Total loci	241
Total categories	19

Comparison between CHM1 and CHM13

CHM1-CHM13	CHM13 intact	CHM13 non-intact	CHM13 absent	Total
CHM1 intact	102	29	23	154
CHM1 non-intact	19	NA	NA	NA
CHM1 absent	23	NA	NA	NA
Total	144	NA	NA	NA

Total LINE-1	298
Total loci	196
Total categories	5

Comparison between GRCh38 and CHM1

CHM1-GRCh38	GRCh38 intact	GRCh38 non-intact	GRCh38 absent	Total
CHM1 intact	95	31	28	154
CHM1 non-intact	24	NA	NA	NA
CHM1 absent	37	NA	NA	NA
Total	156	NA	NA	NA

Total LINE-1	310
Total loci	215
Total categories	5

Comparison between GRCh38 and CHM13

CHM13-GRCh38	GRCh38 intact	GRCh38 non-intact	GRCh38 absent	Total
CHM13 intact	89	27	28	144
CHM13 non-intact	30	NA	NA	NA
CHM13 absent	37	NA	NA	NA
Total	156	NA	NA	NA

Total LINE-1	300
Total loci	211
Total categories	5
