# Supplementary material for: Evolutionary insights from profiling LINE-1 activity at allelic resolution in a single human genome
Source: EMBO J. 2023 Dec 18;43(1):6. doi: 10.1038/s44318-023-00007-y (PMC10883270; doi:10.1038/s44318-023-00007-y)
Supplement: Supplementary file 9 — Expanded View Figures [file 44318_2023_7_MOESM9_ESM.pdf]

Expanded View Figures

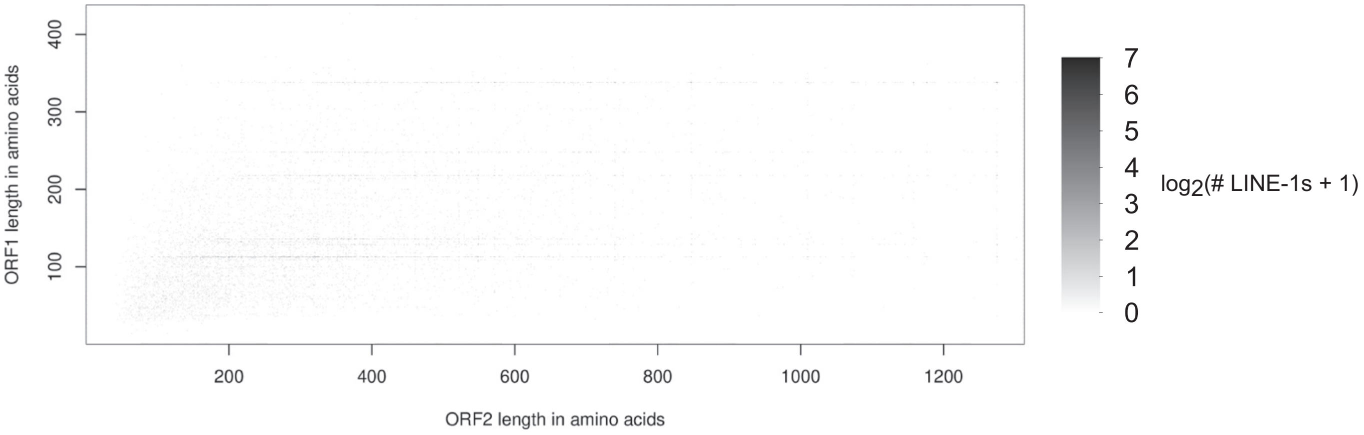

Figure EV1. Heatmap of translated ORF lengths in full-length LINE-1s from CHM1.

A zoomed-out version of Fig. 1C shows the distribution of all called translated ORF lengths in the complete set of full-length (>5000 bp) LINE-1 sequences. Many full-length sequences have either an intact ORF1 or ORF2, but only 148 have both intact ORFs that align along the entire length of a reference amino acid sequence ($L1_{RP}$).

 © The Author(s)

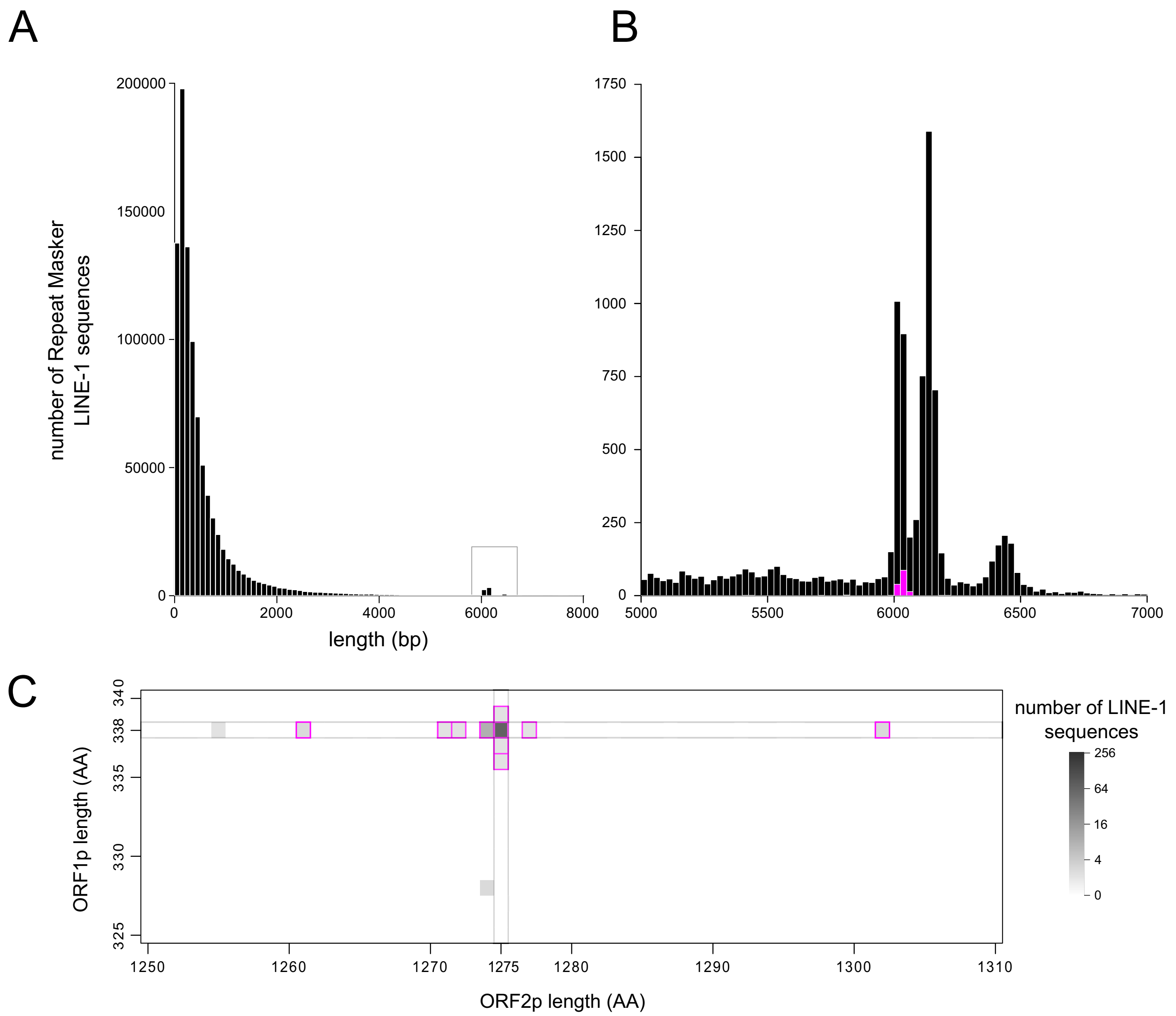

Figure EV2. Identification of intact LINE-1s in CHM1.

(**A**) The distribution of lengths of LINE-1-masked sequences. (**B**) A zoomed-in view of the boxed region of the complete length histogram. Intact LINE-1 sequences are highlighted in magenta. (**C**) A heatmap of the number of sequences with the indicated translated ORF1 and ORF2 lengths. Within the full-length set of LINE-1 sequences, magenta-outlined pixels encode putative ORFs that align along the entire length of the ORF1p and ORF2p sequences of a reference element ($L1_{RP}$).

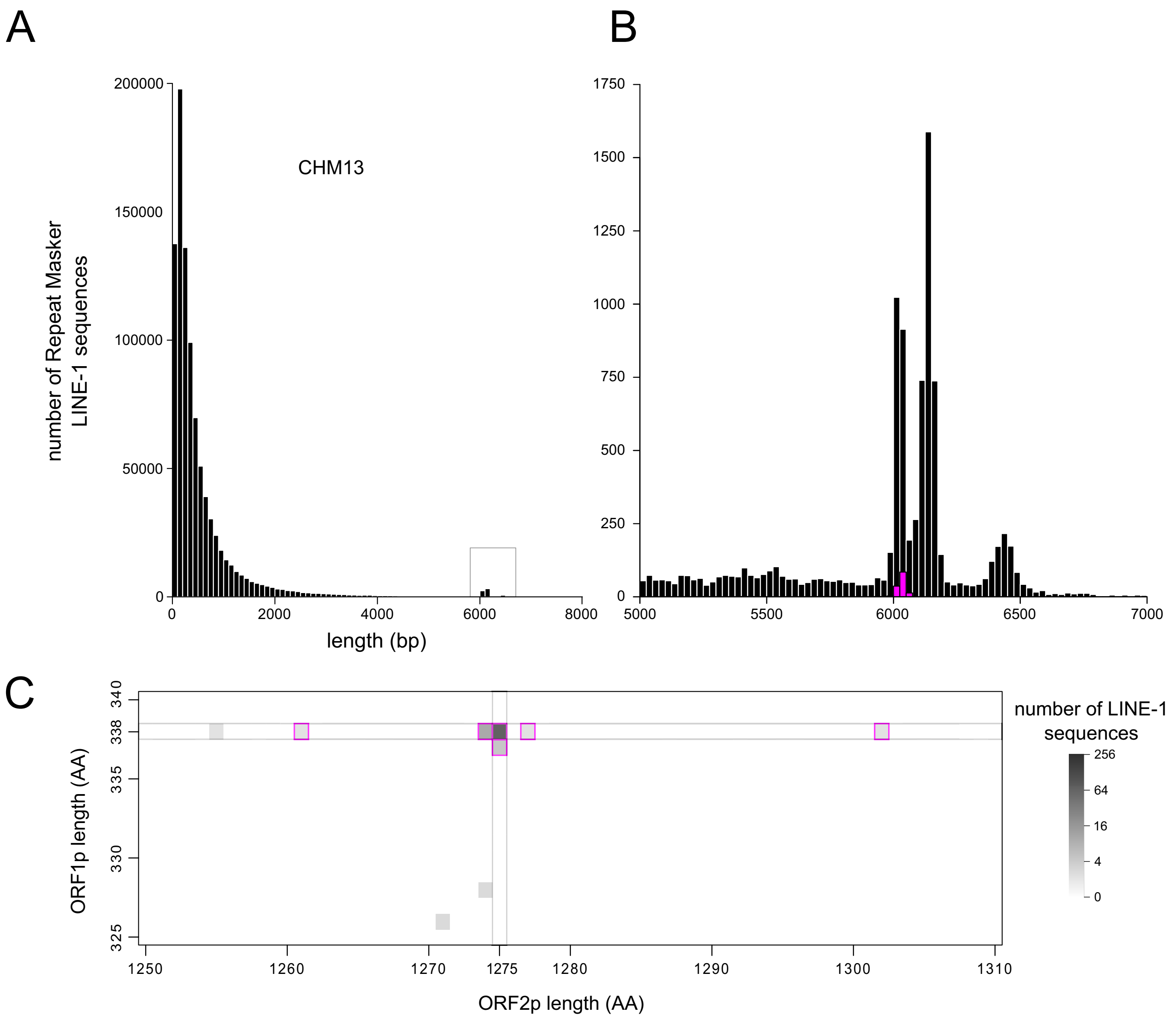

Figure EV3. Identification of intact LINE-1s in CHM13.

(**A**) The distribution of lengths of LINE-1-masked sequences. (**B**) A zoomed-in view of the boxed region of the complete length histogram. Intact LINE-1 sequences are highlighted in magenta. (**C**) A heatmap of the number of sequences with the indicated translated ORF1 and ORF2 lengths. Within the full-length set of LINE-1 sequences, magenta-outlined pixels encode putative ORFs that align along the entire length of the ORF1p and ORF2p sequences of a reference element ($L1_{RP}$).

 © The Author(s)

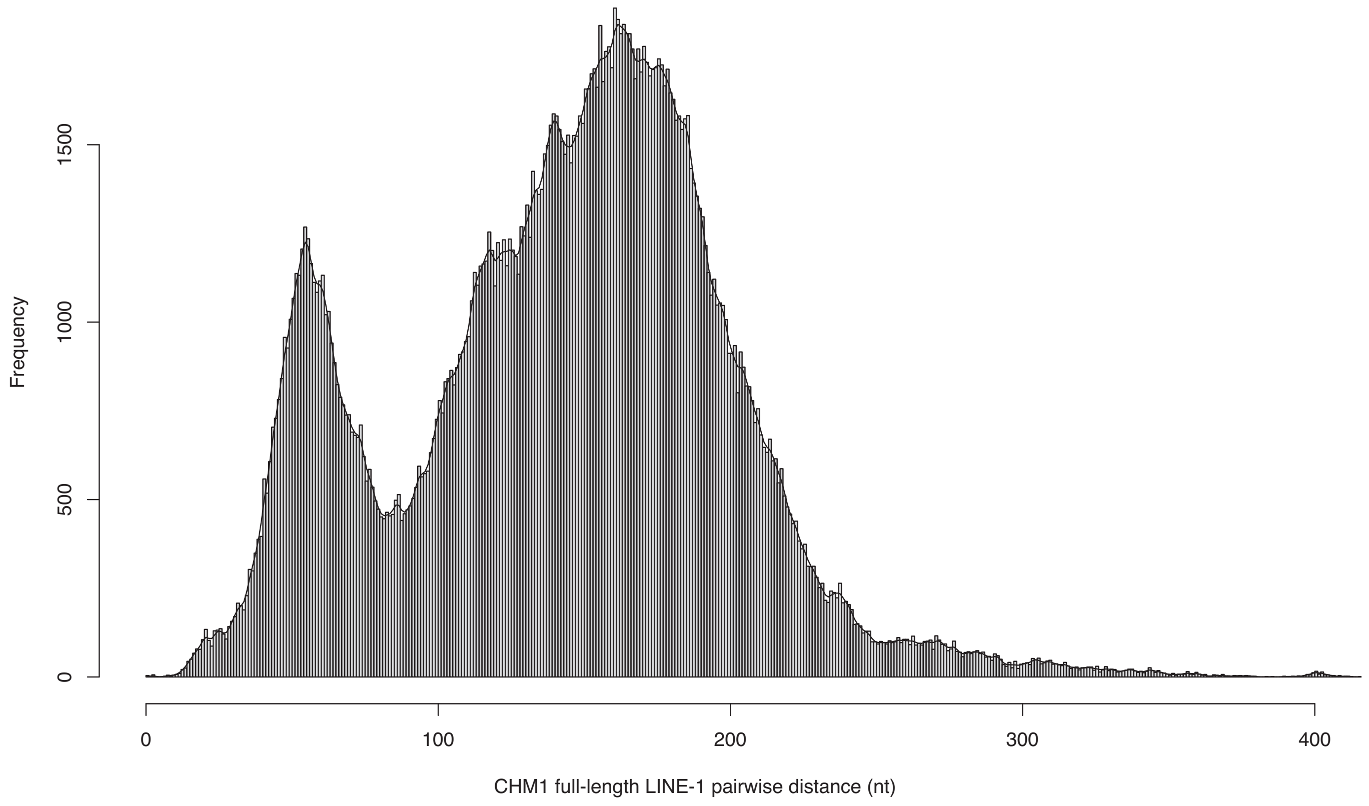

Figure EV4. Distribution of pairwise distances between full-length LINE-1s in CHM1.

The histogram shows the frequency of bins of nucleotide distances for pairwise comparisons of all full-length LINE-1 pairs in CHM1.

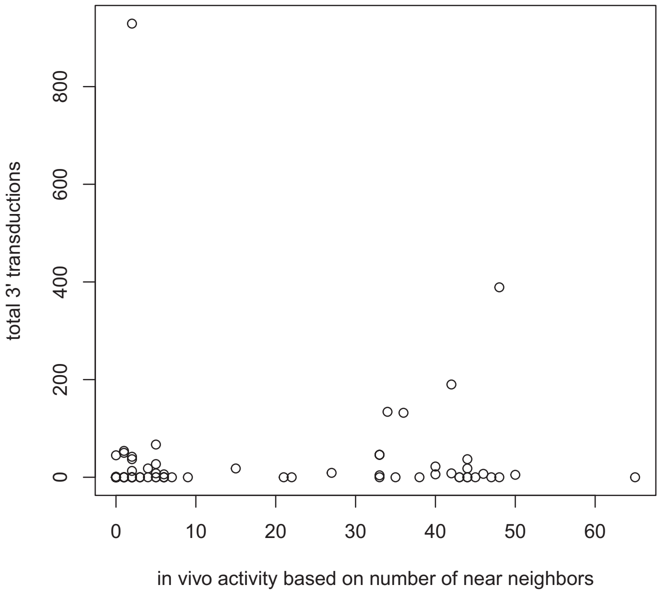

Figure EV5. Scatterplot comparison of in vivo fitness and 3′ transductions of intact CHM1 LINE-1s.

In vivo fitness of a LINE-1 is quantified as the number of full-length LINE-1s within 28 nucleotides distance to that LINE-1. The number of 3′ transductions is the combined number of full-length LINE-1s reported by Tubio et al (2014), Rodriguez-Martin et al (2020), and Chuang et al (2021).

 © The Author(s)
